# The role of infants’ mother-directed gaze, maternal sensitivity, and emotion recognition in childhood callous unemotional behaviours

**DOI:** 10.1007/s00787-017-0967-1

**Published:** 2017-02-28

**Authors:** R. Bedford, N. J. Wagner, P. D. Rehder, C. Propper, M. T. Willoughby, R. W. Mills-Koonce

**Affiliations:** 1 Biostatistics and Health Informatics Department, Institute of Psychology, Psychiatry and Neuroscience, King’s College London, London, England; 2 Human Development and Quantitative Methodology, University of Maryland, College Park, USA; 3 Human Development and Family Studies Department, University of North Carolina-Greensboro, Greensboro, USA; 4 Center for Developmental Science, University of North Carolina-Chapel Hill, Chapel Hill, USA; 5 RTI International, Research Triangle Park, NC USA

**Keywords:** Callous unemotional behaviours, Infant, Face, Sensitive parenting, Emotion recognition

## Abstract

While some children with callous unemotional (CU) behaviours show difficulty recognizing emotional expressions, the underlying developmental pathways are not well understood. Reduced infant attention to the caregiver’s face and a lack of sensitive parenting have previously been associated with emerging CU features. The current study examined whether facial emotion recognition mediates the association between infants’ mother-directed gaze, maternal sensitivity, and later CU behaviours. Participants were 206 full-term infants and their families from a prospective longitudinal study, the Durham Child Health and Development Study (DCHDS). Measures of infants’ mother-directed gaze, and maternal sensitivity were collected at 6 months, facial emotion recognition performance at 6 years, and CU behaviours at 7 years. A path analysis showed a significant effect of emotion recognition predicting CU behaviours (*β* = −0.275, S.E. = 0.084, *p* = 0.001). While the main effects of infants' mother-directed gaze and maternal sensitivity were not significant, their interaction significantly predicted CU behaviours (*β* = 0.194, S.E. = 0.081, *p* = 0.016) with region of significance analysis showing a significant negative relationship between infant gaze and later CU behaviours only for those with low maternal sensitivity. There were no indirect effects of infants’ mother-directed gaze, maternal sensitivity or the mother-directed gaze by maternal sensitivity interaction via emotion recognition. Emotion recognition appears to act as an independent predictor of CU behaviours, rather than mediating the relationship between infants’ mother-directed gaze and maternal sensitivity with later CU behaviours. This supports the idea of multiple risk factors for CU behaviours.

**Electronic supplementary material:**

The online version of this article (doi:10.1007/s00787-017-0967-1) contains supplementary material, which is available to authorized users.

## Introduction

Callous unemotional (CU) behaviours are a proposed precursor of adult psychopathy. Children with CU behaviours are characterized by shallow affect, reduced emotionality, and a lack of feelings of guilt or empathy [30]. Although research with older children demonstrates links between CU behaviours and difficulty recognizing emotional expressions [18], the developmental pathways underlying this association are not well understood. Recent studies have begun the search for antecedents, infant behaviours which are associated with the subsequent development of CU behaviours, and one proposed early CU precursor is reduced attention to the face of a caregiver [6, 16]. Early interactions with caregivers, which play an important role in the emergence of adaptive behavioural and emotional functioning [9, 58], contribute to the consolidation and maturation of socio-emotional systems that underlie later adjustment or maladjustment [12]. In the current study we aimed first to examine the associations between maternal sensitivity in infancy and infants’ mother-directed gaze with later CU behaviours, and second, to test whether emotion expression recognition mediates the associations between infants’ mother-directed gaze, maternal sensitivity, and later CU behaviours.

Early socio-environmental factors, such as parenting, are known to play an important role in the development of CU behaviours [68]. Although the majority of parenting studies have been cross-sectional, several studies have found prospective associations between positive parenting, such as sensitive responding, warmth and positive reinforcement, and lower CU behaviours later in development [5, 6, 32, 46, 66, 77]. Passive and evocative gene–environment correlations, whereby parents who are more sensitive and warm have children with lower CU behaviours, are always difficult to rule out. However, Waller et al. [70] used an adoptive design to show that lower levels of parent reinforcement were related to higher CU behaviours in parents who were not genetically related to their children (also see [33]). This provides strong evidence that aspects of sensitive parenting in early life contribute to lower levels of CU behaviours.

One mechanism through which parenting may play a role in the development of social-emotional problems characteristic of callous unemotional behaviours is emotional understanding [11]. It is well established that adult psychopaths show impaired emotion recognition abilities and, although the most commonly reported difficulty is in recognising fearful facial expressions, a recent meta-analysis found impairments across a range of emotions [18]. Blair [7] suggested that a failure to recognise distress cues in others may play a role in atypical empathy development. This has been supported by the finding that children with CU behaviours show difficulties in recognising facial affect, particularly for negative emotions such as fear, sadness, and anger (e.g., [8, 17, 29, 49, 60]. Dadds et al. [16] expanded this idea, arguing that impaired emotion recognition may reflect more general deficits in attention to social-emotional stimuli. They suggest that the developmental pathway to CU traits may actually be via reduced emotional engagement and attention to caregivers.

Attention to the face, and specifically the eye region, has been identified in the literature as a potential contributor to the emergence of emotion recognition, particularly for the recognition of fearful facial expressions [13, 15–17]. In the current paper we suggest that emotion recognition may serve as one potential mechanism through which early attention to caregivers influences the development of CU behaviours. In infancy, increased face preference at just 5 weeks of age has been shown to predict lower CU behaviours later in development [6]. Additionally, using data from the current sample, Wagner and colleagues found that high levels of gaze toward the mother may protect against the development of CU behaviours, particularly for infants who show deficits in normative patterns of reactivity early in life [67]. The current study extends this work by including CU outcomes later in childhood and by examining the mediating role of emotion recognition. Although previous studies suggest clear links between attention to the face, emotion recognition, and CU traits, establishing whether emotion recognition mediates the relationship between infant attention to the face and later CU traits is of particular importance, given the ability to intervene on this behaviour [14].

In addition to the independent contributions of early attention to the face and sensitive parenting to lower CU behaviours, there is both theoretical and empirical support for these factors eliciting an interactive influence on the development of CU behaviours [16]. Infants’ visual orientation is an important mechanism of communication with caregivers, promoting affiliation and continued reciprocity that, when responded to sensitively by the parent, contributes to the formation of secure attachment relationships [2, 9]. Interestingly, in addition to deficits in eye-contact, children with CU behaviours are more likely to form insecure attachments than youth with normative levels of behaviour problems and CU behaviours [26, 52, 53]. Although Bedford et al. [6] did not find evidence for an interaction between maternal sensitivity and face preference, work by Dadds et al. [13, 16]—which found reduced eye contact in high CU-behaviour boys during interactions with their parents, despite the fact that mothers showed typical levels of eye-contact—provides support for links between early dyadic processes and the development of CU behaviours. It is clear that sensitive parenting and infant attention to caregivers serve to enhance the positive outcomes associated with adaptive parenting [35] and buffer against maladaptive trajectories of antisocial behaviours [39]. As such, the current study will also test the extent to which maternal sensitivity and infants’ mother-directed gaze interactively predict later CU behaviours.

In the current study we use a large, community-based, longitudinal sample to test (1) whether infants’ mother-directed gaze, maternal sensitivity, the interaction between infant gaze and maternal sensitivity, and childhood emotion recognition predict later CU behaviours; and (2) whether emotion recognition ability mediates the association of infant gaze and maternal sensitivity with later CU behaviours.

## Method

### Participants

The current study used participants from the Durham Child Health and Development Study (DCHDS), a prospective longitudinal study of 206 full-term infants and their families who were recruited when the children were 3 months of age (see Table 1). The sample was 57% African American and 43% European American, and approximately 53% of families were low income (below 200% of the federal poverty level). This study was approved by the Institutional Review Board at the University of North Carolina at Chapel Hill.

**Table 1:** Descriptive statistics and bivariate correlation matrix

	1	2	3	4	5	6	7	8	9
CU behaviours, ICU (7 years)	–								
Emotion recognition (6 years)	−.29*	–							
Infant gaze (6 months)	−.02	−.02	–						
Maternal sensitivity (6 months)	−.21*	0.13	−.03	–					
CU behaviours, CBCL (2.5 years)	0.35*	−.07	0.05	−.16*	–				
Mothers’ neutral exp. (6 months)	0.03	0.04	−.16	−.13	0.09	–			
Income (6 months)	0.01	0.20*	−.19*	0.26*	−.03	−.02	–		
Sex (0 = male; 1 = female)	−.08	0.09	0.06	−.06	−.10	0.12	−.02	–	
Ethnicity (0 = EA; 1 = AA)	−.02	0.07	0.22*	−.31*	0.004	0.08	−.26*	0.08	–
Number	132	130	165	175	178	151	178	206	206
Mean	0.71	0.79	0.4	3.29	1.08	0.31	3.03	0.49	0.57
Standard deviation	0.32	0.18	0.22	0.81	1.34	0.26	2.67	0.50	0.50

*ICU* inventory of callous unemotional traits, *CBCL* child behaviour checklist, *AA* African American, *EA* European American

* *p* values <0.05

### Procedure and measures

The current study uses data collection from the 6-month and 2.5-, 6-, and 7-year time points. Information on children’s sex and race was collected upon entry into the study. All ratings and observations occurred in a laboratory setting, except for the observation of parent–child interactions during free play, which were conducted at the participants’ homes. At each visit, infants and their mothers participated in a number of joint and individual activities and mothers completed a standardized interview and demographic questionnaires. Transportation was provided to families who required assistance getting to and from the laboratory.

#### Infant and parent gaze

The infants were observed in the Face-to-Face Still-Face Paradigm (FFSFP; [3, 63] during the 6-month lab visit to assess infant behaviours, specifically mother-directed gaze. Mothers placed infants in an infant chair on a table and situated themselves in a chair that was placed directly in front of the infants’ chair. Mothers were given a set of standardized instructions for each episode of the FFSFP (i.e., face-to-face, still-face, reunion). During the face-to-face episode, mothers were instructed to play with their babies as they normally would for 2 min. Then, mothers were told to turn away from their infant for 15 s, and then to turn back toward their infants for the still-face episode. Mothers were to look at their infant for 2 min without providing any verbal or facial response to the infant (i.e., maintaining a still face). Only behaviour during the initial face-to-face episode was analysed in the current study. The FFSFP was stopped if the infant was unable to be soothed at any point during the procedure. The episodes were video recorded using a split-screen procedure to ensure that the behaviours of both mothers and infants could be observed during the entire interaction.

Infants’ and mothers’ gaze during the FFSFP episodes was coded by trained coders. In separate viewings of the videotapes, different research assistants coded infants’ and mothers’ direction of gaze in one second intervals. Gaze was coded as toward or away from the partner. Coders were initially trained to reliability using pre-existing video-recorded FFSFP interactions. Inter-observer agreement was determined by randomly selecting 15% of the interactions to be coded by a second coder. The coders were considered to be in agreement if they coded the same behaviour within one second of each other. Reliability was calculated using kappa to correct for chance agreement (see [47] for more information about this coding procedure). Overall, coders reliably identified mother affect (*K* = 0.83), infant affect (*K* = 0.89), infants’ direction of gaze (*K* = 0.90), and mothers’ direction of gaze (*K* = 0.85). Only data from the face-to-face interaction was analysed in the current study. The primary measure of interest which has been validated in multiple studies (e.g., [47, 48, 67] was infants’ gaze toward the mother’s face during the face-to-face interaction computed as a proportion of the total valid interaction time. Mothers’ affect during the face-to-face interaction was included as a covariate.

#### Maternal sensitivity

Mothers and their infant were observed during a free-play task lasting 10 min as part of the home visit completed when the infants were 6 months of age. A set of standard toys were arranged on a blanket and the mothers were asked to play with their infants as they normally would on a typical day. All interactions were videotaped and later viewed by trained and reliable coders who rated the interactions using 5-point subscales (measures adapted from the [51]. Previous factor analysis supported the creation of a maternal sensitivity composite which included measures of sensitivity, detachment (reverse-scored), stimulation of development, positive regard, and animation (see Willoughby et al. [72–74]). Each coding team consisted of four to five coders and included one or two master coders. Each coder was trained to be reliable with the master coder(s), and ongoing reliability was calculated using intraclass correlations across coding pairs for each parenting subscale. Reliability across subscales and composites was high (intraclass correlations >0.80).

#### Emotion recognition

Children’s emotion identification was assessed using the facial expressions subscale of the Assessment of Children’s Emotional Skills (ACES; [56] administered at the 6-year visit. In the facial expressions section, children were presented with still photographs of elementary-aged children posing various facial expressions. For each photograph, the administrator asked, “Does he/she feel happy, sad, mad, scared, or no feeling?” and recorded the child’s response. There were eight emotion trials (2× happy, sad, mad, and scared) which were scored correct or incorrect, and a total score was computed. There were also and eight ambiguous trials, where the expression did not clearly show any one emotion; however, for the current study only responses from the eight emotion recognition trials were analysed.

#### Callous unemotional behaviours

The inventory of callous unemotional traits (ICU; [28] was used to assess CU behaviours when children were 7 years old (Cronbach’s alpha = 0.83). The ICU was completed by maternal primary caregivers who responded to 24 items on a 4-point Likert scale ranging from 0 (not at all true) to 3 (definitely true). After reverse coding the items based [34] we calculated Cronbach’s alpha, which suggested that item 10 was loaded in the opposite direction to all other items, so it was also reverse coded (range of scores 2–39). However, we also reran the analysis removing items 2 and 10, based on Kimonis et al. [34], see supplementary materials. The items, which are comprised of highly established clinical assessments (e.g., APSD, PCL-YV) and include questions about the extent to which the child uses emotions, expresses feelings, cares about getting in trouble, seems cold and uncaring, and hurts others’ feelings, demonstrate acceptable factor structure and predictive utility with samples ranging in age from 13 to 20 years of age (see [23, 25, 34, 55]), and with samples as young as age 3 (see [24]).

#### Additional covariates

Child’s sex and race were collected at the time of recruitment. Poverty status was collected at the 6-month home visit and determined using federal guidelines. Earlier CU behaviours were derived from the Achenbach System of Empirically Based Assessment, Preschool Forms (ASEBA; [1]), which was completed by primary caregivers at the 2.5-year visit. This standardized assessment indexes children’s behavioural and emotional problems using caregivers’ ratings of their child’s behaviour currently or within the past 2 months [1]. Data from the current study [75] and data from the NICHD Study of Early Child Care and Youth Development [76], suggest that five items drawn from the ASEBA (“no guilt after misbehave”, “punish doesn’t change behaviour”, “unresponsive to affection”, “shows little affection”, and “too little fear”) can reliably be used to measure individual differences in CU behaviours at these early ages. Sex, race, income, and earlier CU behaviours were included as covariates in each analytic model.

### Statistical analysis

Path analysis models were used to test the extent to which maternal sensitivity, infants’ mother-directed gaze, and their interaction predicted mother-rated CU behaviours at 7 years, and to establish whether children’s 6-year emotion recognition difficulties mediated these associations. We first ran a model including the direct effects of maternal sensitivity and infants’ mother-directed gaze on later emotion recognition and CU behaviours, with an indirect pathway from infant predictors to later CU behaviours via emotion recognition, controlling for mother’s neutral affect, early CU behaviours (at 30 months), ethnicity, sex, and poverty status. Next, to test whether maternal sensitivity moderates the hypothesized links between infants’ mother-directed gaze and later CU behaviours, we re-ran the model to include the interaction effect of infants’ mother-directed gaze by maternal sensitivity on later emotion recognition and CU behaviours. Significant interactions were probed using the online utility and computational tools for probing two-way interaction effects in multiple linear regressions [54]. Following Roisman et al. [54], we ran region of significance analyses. Exogenous variables were allowed to covary, and all predictors and outcomes were centred to aid interpretation. Missing data were handled using the full information maximum likelihood methods [22]. All path analyses were estimated using Mplus [50] and descriptive statistics were obtained using STATA [59]. Standardized model results are reported.

## Results

Descriptive statistics and correlations between covariates and variables of interest are presented in Table 1. The correlation matrix showed significant associations between increased CU behaviours at 7 years and reduced 6-year emotion recognition (*r* = −0.29), lower 6-month maternal sensitivity (*r* = −0.21), and higher early 30-month CU behaviours (*r* = 0.35). Emotion recognition was not significantly correlated with either maternal sensitivity or infant mother-directed gaze.

**Fig. 1:**
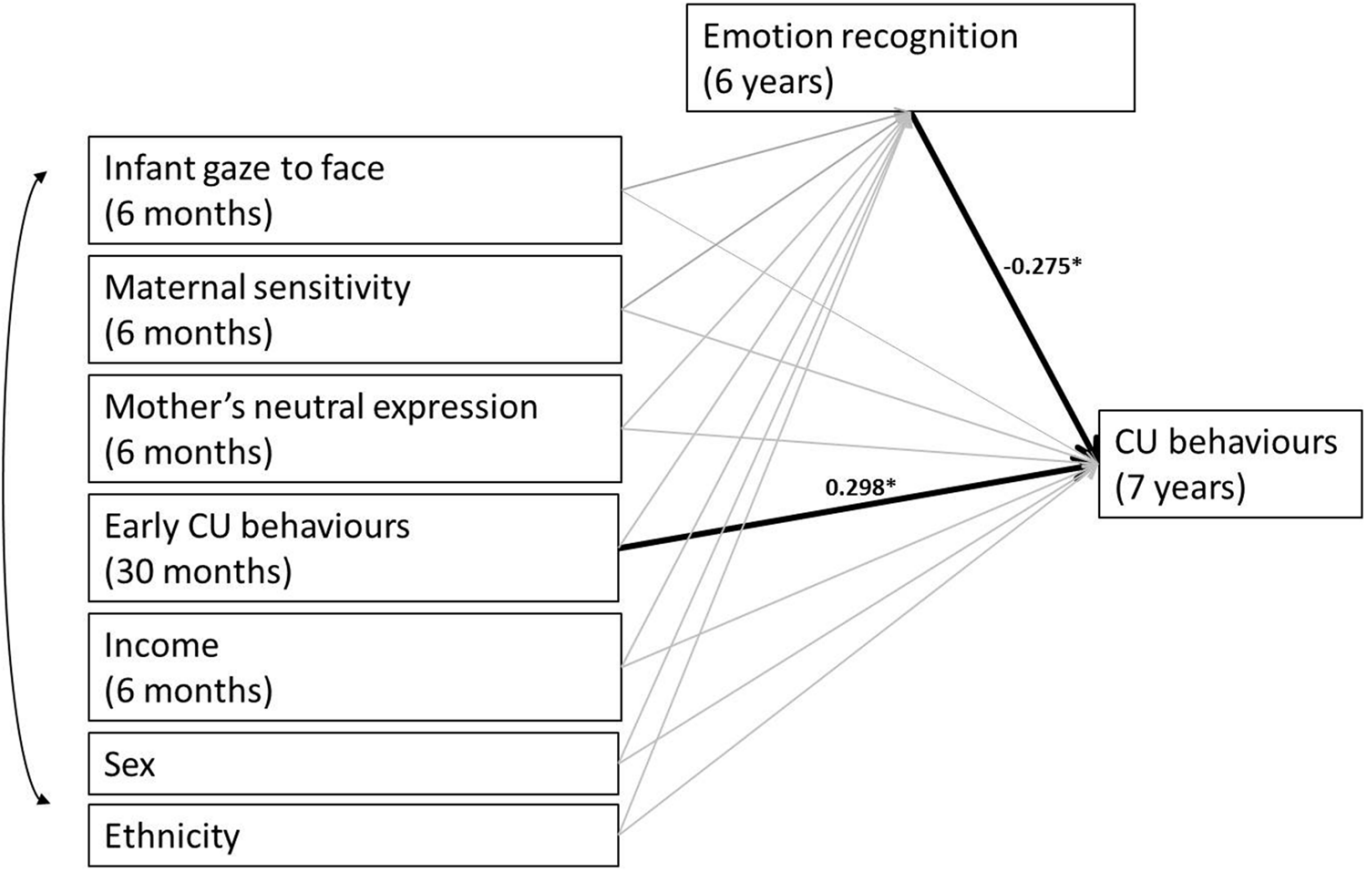
Path diagram showing the significant effect of emotion recognition accuracy at 6 years in the prediction of 7-year *CU behaviours*

A saturated path analysis model was estimated using a maximum likelihood estimator (see Fig. 1; Table 2). Emotion recognition at 6 years significantly predicted 7-year CU behaviours, *β* = −0.275, S.E. = 0.084, *p* = 0.001, with poorer emotion recognition ability associated with higher CU behaviours. The effect of maternal sensitivity was only marginally significant, *β* = −0.155, S.E. = 0.086, *p* = 0.072, and contrary to our hypothesis, there was no main effect of infants’ mother-directed gaze, *β* = −0.039, S.E. = 0.09, *p* = 0.66. Children’s early CU behaviours at 2.5 years significantly predicted later CU at age 7, *β* = 0.298, S.E. = 0.078, *p* < 0.001, but none of the other covariates reached significance (*p* values >0.432). The model accounted for 22.1% of the variance in CU traits. No significant associations were found between infants’ mother-directed gaze, *β* = 0.033, S.E. = 0.10, *p* = 0.739, or maternal sensitivity, *β* = 0.076, S.E. = 0.095, *p* = 0.420, and later emotion recognition, and none of the covariates significantly predicted emotion recognition (*p* values >0.081). Finally, no significant indirect effects were found for either infants’ mother-directed gaze, *β* = −0.013, S.E. = 0.040, *p* = 0.741, or maternal sensitivity, *β* = −0.008, S.E. = 0.011, *p* = 0.433, to CU behaviours via emotion recognition. Although we controlled for sex in the analysis, we also tested whether sex moderates the association between infant measures and later CU behaviours, finding a marginal interaction for maternal sensitivity by sex (see supplementary materials).

**Table 2:** Standardised model results

	Model 1		Model 2	
	*β* (S.E)	95% CI	*β* (S.E)	95% CI
CU behaviours (ICU)—7 years				
Emotion recognition	−0.275* (0.084)	−0.44, −0.11	−0.293* (0.083)	−0.46, −0.13
Infant gaze	−0.039 (0.090)	−0.22, 0.14	−0.004 (0.089)	−0.18, 0.17
Maternal sensitivity	−0.155 (0.086)	−0.32, 0.01	−0.129 (0.086)	−0.30, 0.04
CU behaviours (CBCL)	0.298* (0.078)	0.15, 0.45	0.331* (0.077)	0.18, 0.48
Mothers’ neutral expression	−0.016 (0.084)	−0.18, 0.15	0.004 (0.083)	−0.16, 0.17
Income	0.072 (0.092)	−0.11, 0.25	0.087 (0.090)	−0.09, 0.26
Sex	−0.057 (0.080)	−0.21, 0.10	−0.047 (0.079)	−0.20, 0.11
Ethnicity	−0.062 (0.088)	−0.24, 0.11	−0.074 (0.087)	−0.24, 0.10
Infant gaze × maternal sensitivity	–	–	0.194* (0.081)	0.04, 0.35
Emotion recognition—6 years				
Infant gaze	0.033 (0.100)	−0.16, 0.23	0.061 (0.100)	−0.14, 0.26
Maternal sensitivity	0.076 (0.095)	−0.11, 0.26	0.084 (0.094)	−0.10, 0.27
CU behaviours (CBCL)	−0.049 (0.092)	−0.23, 0.13	−0.028 (0.093)	−0.21, 0.15
Mothers’ neutral expression	0.072 (0.094)	−0.11, 0.26	0.089 (0.093)	−0.09, 0.27
Income	0.177 (0.101)	−0.02, 0.38	0.187 (0.100)	−0.009, 0.38
Sex	−0.015 (0.088)	−0.19, 0.16	−0.012 (0.087)	−0.18, 0.16
Ethnicity	0.033 (0.100)	−0.16, 0.23	0.025 (0.099)	−0.17, 0.22
Infant gaze × maternal sensitivity	–	–	0.122 (0.090)	−0.06, 0.30

**Fig. 2:**
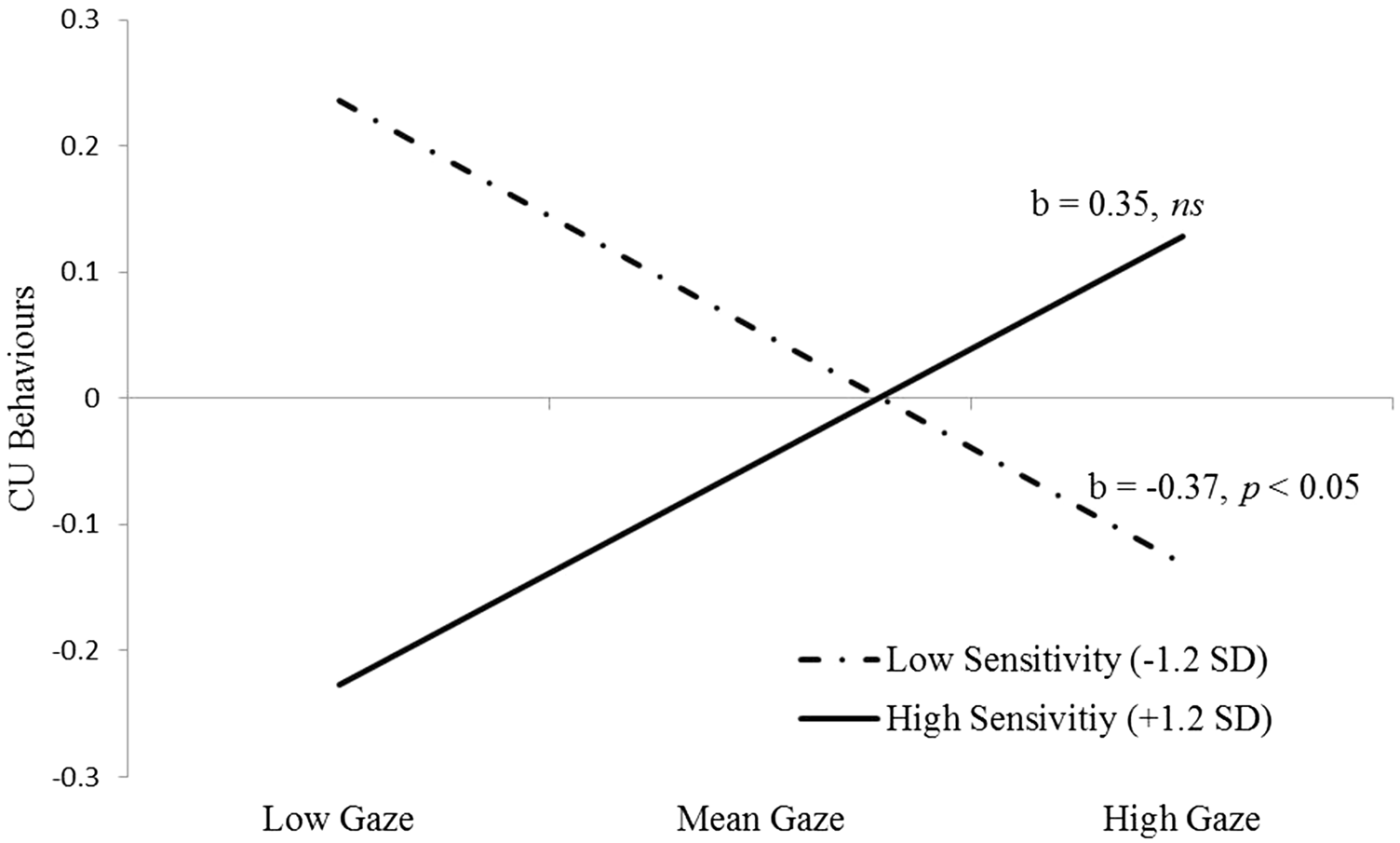
The interaction between 6-month-olds’ mother-directed gaze and maternal sensitivity in the prediction of *CU behaviours* at 7 years of age; simple slopes maternal sensitivity are plotted at the RoS for *low sensitivity* (i.e., +/-1.2 SDs)

Next, we re-ran the model adding the interaction between infants’ mother-directed gaze by maternal sensitivity as a predictor of emotion recognition and CU behaviours. While there was no significant effect of the mother-directed gaze by maternal sensitivity interaction on emotion recognition, *β* = 0.122, S.E. = 0.090, *p* = 0.178, there was a significant interaction effect in the prediction of later CU behaviours, *β* = 0.194, S.E. = 0.081, *p* = 0.016. Region of significance (RoS) and simple slopes analysis showed that the association between infants’ mother-directed gaze and CU behaviours was significant only at low levels of infant gaze (scores below 0.03, i.e., <0.14 SDs below the mean, simple slope at −1 SD = −0.22, *t*(196) = 3.19, *p* = 0.002), and for maternal sensitivity scores below −1.0 (i.e., <1.2 SDs below the mean), simple slope at −1 SD = −0.81 is −0.30, *t*(196) = 1.81, *p* = 0.072 (see Fig. 2 for a plot of the simple slopes at the RoS). In other words, there was a significant negative association between infant gaze and later CU behaviours only for those with low levels of maternal sensitivity. The *R* ^2^ value for CU traits in Model 2 was 25.3%.

## Discussion

The current study tested whether early maternal sensitivity and infants’ mother-directed gaze independently and interactively predict later CU behaviours, as well as the extent to which deficits in emotion recognition mediate these associations. Although the main effect of infant gaze was not significant, and maternal sensitivity was only a marginally significant predictor of later CU behaviours, we found evidence for an interaction between 6-month-olds’ gaze to their mother’s face and maternal sensitivity in the prediction of CU behaviours at 7 years of age. Infants with low levels of gaze to the parent’s face during a face-to-face interaction showed higher levels of CU behaviours at age 7 years, only when maternal sensitivity was low. Further, we found that lower emotion recognition accuracy at 6 years directly predicted later CU behaviours, but we did not find associations of infants’ mother-directed gaze or maternal sensitivity with emotion recognition, nor an indirect pathway to CU behaviours. Emotion recognition thus appears to act as an independent predictor, rather than mediating the relationship between infants’ mother-directed gaze and maternal sensitivity with later CU behaviours, consistent with the idea of additive contributions of multiple risk factors to the development of psychopathology [20, 62].

Our results showed a significant longitudinal association between emotion recognition difficulties and CU behaviours, with poorer emotion recognition performance at 6 years associated with higher CU behaviours at 7 years, controlling for earlier CU behaviours. This finding is consistent with the literature showing impaired emotion recognition in children with CU behaviours (e.g., [18, 29]). One hypothesised mechanism to explain this association is attention to emotional expressions, specifically distress cues, which is known to be important in the development of empathic responses and conscience, and may deter malicious acts [7, 57]. Concurrent attention to the eye region of faces plays an important role in emotion recognition, particularly for fear recognition. Indeed, fear recognition deficits in children with high CU behaviours abate following instruction to attend to the eyes [4]. Contrary to our hypothesis, we did not find a significant association between infant attention to the face and later emotion recognition performance, nor any indirect effects. It is possible that concurrent attention during an emotion recognition task is more important than the longitudinal impact of infants’ attention to the face 5 years earlier. There are several other possible explanations of these results to consider. First, our infant measure is video-coded attention to the face, and it may be that a more fine-grained measure of attention to the eyes, using real-time eye-tracking, would show significant associations with later emotion recognition. It is also possible that attention to the face during this face-to-face episode, where parents may or may not be emotionally expressive, is not generalizable to the wider context of infant’s attention to emotional expressions.

Parents’ early sensitivity and emotion socialization practices contribute to conscience development, influence the formation of attachment relationships, and have implications for children’s abilities to understand and recognize emotion [21, 40, 42, 43, 45], all of which may play a role in the development of CU behaviours [11, 41]. Despite an increasing number of studies demonstrating links between early sensitivity and eventual CU behaviours [46, 66, 69, 72], and work suggesting that the influences of early sensitivity on later CU behaviours are mediated through children’s emotional understanding [11], the current study found no support for the hypothesis that maternal sensitivity at 6 months predicts CU behaviours via its influences on children’s emotion recognition. This is consistent with an RCT in children with high CU traits which showed no mediating effect of emotion recognition [14], although emotion recognition training did lead to improvements in affective empathy and conduct problems in these children. There are also a diversity of mechanisms through which parents socialize children’s emotional capacities, and it is possible that the measure of maternal sensitivity used in the current study does not capture specific emotion-related practices relevant for the tested processes. For example, Eisenberg et al. [21] suggest that parents’ reactions to children’s emotional expressions, parents’ emotional expressiveness, and parent–child discussion of emotion are primary ways in which children’s emotional competencies are supported. Extant literature shows that emotion language use at 15 months is positively associated with emotion understanding in toddlerhood [61]. Mothers of children demonstrating elevated CU behaviours have been shown to use emotion socialization practices that dismiss or devalue children’s emotions (Pasalich et al. [52, 53], and Centifanti and colleagues [11] found that mental-state language at 8 months indirectly predicted children’s CU behaviours through emotion understanding. Future studies which incorporate a diversity of emotion socialization experiences in infancy may identify mediated pathways to CU behaviours through emotion recognition.

Consistent with the idea of a dynamic interplay between infants’ early behaviour and their environment in the development of CU behaviours, we also found that the interaction between infants’ mother-directed gaze and maternal sensitivity played an important role in the development of later CU behaviours. Further, as the main effects of infants’ mother-directed gaze and maternal sensitivity did not reach significance, our results suggest that the relationship between infants’ mother-directed gaze and CU behaviours is dependent on the level of maternal sensitivity in our sample. The interaction effect was driven by low maternal sensitivity, such that reduced levels of infants’ mother-directed gaze was only a risk factor for later CU behaviours in the context of low maternal sensitivity.

Early dyadic interactions between infants and their caregivers are often discussed in terms of synchronization and play a distinct role in the emergence of behavioural and emotional regulation and healthy attachment relationships [58, 71]. An infant’s propensity for engaging in dyadic, or synchronized, interactions with a caregiver may have important implications for the development of CU behaviours, especially given their association with insecure attachment relationships [52] and disrupted emotional bonds [27]. Our findings suggest that these fundamental developmental processes may be undermined by infants’ reduced gaze toward their caregiver during interactions, but only in the context of caregiving characterized by a lack of sensitivity and warmth. As suggested by the Mutual Regulation Model (MRM; [64], mother–infant dyads are jointly regulated toward reciprocal interaction through behavioural and affective feedback, a process which is supported by consistent maternal sensitivity and support [36, 37, 65]. The joint contribution of a lack of infants’ mother-directed gaze and low maternal sensitivity likely undermines the establishment of mutually responsive behaviour, resulting in a cascade of emotional and regulatory difficulties and subsequent CU behaviours.

The current study has several key strengths, which include a longitudinal design, diverse observational measurement, and the inclusion of earlier CU behaviours as a covariate in path analyses. Although this study contributes to our understanding of the development of CU behaviours, future research should examine the possibility of bidirectional associations between infant and parent behaviours. Waller and colleagues found that CU behaviours predicted less maternal sensitivity in toddlerhood [69], and insensitive and harsh parenting behaviours in very early life may inhibit infants’ willingness to openly respond to parents’ attempts to engage [31, 38]. Infants may learn to avert attention away from a chronically insensitive parent as a form of coping [19, 44], a process associated with the formation of insecure attachment relationships [10]. One important limitation of the current method is the relatively blunt measure of emotion recognition. Given that we know that the distribution of attention to the eyes versus mouth regions differs in children with CU behaviours and impacts recognition ability [16], having eye-tracking data would be helpful. In addition, collecting a higher number of emotion recognition trials, looking at a wider range of different expressions, would make it possible to test whether specific emotions (namely fear and distress cues) mediate the association between infant attention to the face and later CU behaviours.

The results of the current study provide evidence for the longitudinal prediction of childhood CU behaviours by earlier emotion recognition abilities at 6 years. Further, while no significant associations were found between 6-month mother-directed gaze or maternal sensitivity and later CU behaviours, their interaction was significant, with reduced infant gaze associated with higher CU traits only for those with low levels of maternal sensitivity. No indirect effects via emotion recognition were found, with emotion recognition acting as an independent predictor of CU behaviours, rather than mediating the relationship between infants’ mother-directed gaze, maternal sensitivity, and later CU behaviours. In addition to highlighting the benefit of incorporating the measurement of multiple influences in infancy, including both parent and child behaviours, the current study emphasises the importance of considering how multiple risk factors work both directly and interactively to predict emerging CU behaviours.

## Electronic supplementary material

Below is the link to the electronic supplementary material.

Supplementary material 1 (DOCX 18 kb)
