# Supplementary material for: The role of infants’ mother-directed gaze, maternal sensitivity, and emotion recognition in childhood callous unemotional behaviours
Source: Eur Child Adolesc Psychiatry. 2017 Feb 28;26(8):947–56. doi: 10.1007/s00787-017-0967-1 (PMC5532412; doi:10.1007/s00787-017-0967-1)
Supplement: Supplementary file 1 — Supplementary material 1 (DOCX 18 kb) [file 787_2017_967_MOESM1_ESM.docx]

Supplementary information

*Removing items 2 & 10 (Kimonis et al., 2008)*

Based on their factor analysis, Kimonis et al. (2008) suggested removing items 2 & 10. To allow comparability across samples we also reran the analysis removing these items from the Inventory of Callous Unemotional traits (ICU, Frick, 2004). Total scores from the remaining 22 items ranged from 0 – 35 in our sample. Results remained substantively similar. Model 1 showed a significant association between emotion recognition and later CU behaviours, *β* = -0.262, *S.E.* = 0.085, *p* = 0.002. The effect of maternal sensitivity did not reach significance, *β* = -0.146, *S.E.* = 0.086, *p* = 0.09, and contrary to our hypothesis, there was no main effect of infants’ mother-directed gaze, *β* = -0.026, *S.E.* = 0.09, *p* = 0.77. Children’s early CU behaviours at 2.5 years significantly predicted later CU at age 7, *β* = 0.304, *S.E.* = 0.078, *p* < 0.001, but none of the other covariates reached significance (*p-values* > 0.477). Again, there were no significant associations between emotion recognition and maternal sensitivity, *β* = 0.077, *S.E.* = 0.095, *p* = 0.42, gaze to the parent, *β* = 0.034, *S.E.* = 0.10, *p* = 0.74, or any covariates (*p values* > 0.083).

In Model 2, the interaction between maternal sensitivity and infant gaze in predicting CU behaviours remained significant, *β* = 0.199, *S.E.* = 0.081, *p* = 0.014. Region of significance (RoS) and simple slopes analysis showed that the association between infants’ mother-directed gaze and CU behaviours was significant *only* at low levels of gaze (scores below -0.03, i.e., <0.14 SDs below the mean; simple slope at -1 SD = -.022, -0.13, *t*(196) = 3.16, *p* = 0.002) and for maternal sensitivity scores below -1.1 (i.e., < 1.4 SDs below the mean), simple slope at -1 SD = -0.81 is -0.29, *t*(196) = 1.71, *p* = 0.089, and above 1.3 (i.e., >1.6 SDs above the mean) simple slope at +1 SD = 0.81 is 0.32, t(196) = 1.63, p = 0.1. This suggests that at low levels of gaze, low parent sensitivity is associated with higher CU behaviours, and low gaze with high sensitivity is associated with lower CU behaviours.

*Sex differences*

The prevalence of CU is higher in males (Essau, Sasagawa & Frick, 2006), and the majority of studies focus predominately or exclusively on male participants. Large, community-based samples, such as in the current study, enable us to directly investigate differential sex effects and several studies have found an the association between positive parenting characteristics, such as maternal sensitivity, and lower CU behaviours only in girls (Barker, Oliver, Viding, Salekin & Maughan, 2011; Bedford et al., 2015; Hawes, Dadds, Frost & Hasking, 2011).

To test whether there were sex differences in the association between infant gaze, maternal sensitivity and later CU behaviours, we additionally added sex by infant gaze, and sex by maternal sensitivity interactions into Model 1. Neither interaction reached significance (infant gaze: *β* = 0.005, *S.E.* = 0.083, *p* = 0.95; maternal sensitivity: *β* = -0.14, *S.E.* = 0.078, *p* = 0.07). However, given the marginal effect for the maternal sensitivity by sex interaction, we broke this down to test the simple slopes. The association between maternal sensitivity and CU behaviours was significant only for the girls (simple slope at 1SD = -0.11, p = 0.01), suggesting that higher maternal sensitivity is associated with lower CU traits in girls.

*References*

Barker, E., Oliver, B., Viding, E., Salekin, R., Maughan, B. (2011). The impact of prenatal maternal risk, fearless temperament, and early parenting on adolescent callous-unemotional traits: A 14-year longitudinal investigation. *J Child Psychol Psychiatry 52,* 878–888.

Bedford, R., Pickles, A., Sharp, H., Wright, N., & Hill, J. (2015). Reduced face preference in infancy: A developmental precursor to callous-unemotional traits? *Biological psychiatry*, *78*(2), 144-150.

Essau, C. A., Sasagawa, S., & Frick, P. J. (2006). Callous-unemotional traits in a community sample of adolescents. *Assessment*, *13*(4), 454-469.

Hawes, D. J., Dadds, M. R., Frost, A. D., & Hasking, P. A. (2011). Do childhood callous-unemotional traits drive change in parenting practices? *Journal of Clinical Child & Adolescent Psychology*, *40*(4), 507-518.

Kimonis, E. R., Frick, P. J., Skeem, J. L., Marsee, M. A., Cruise, K., Munoz, L. C., ... & Morris, A. S. (2008). Assessing callous–unemotional traits in adolescent offenders: Validation of the Inventory of Callous–Unemotional Traits. *International journal of law and psychiatry*, *31*(3), 241-252.
